# Hepatitis delta virus RNA decline post-inoculation in human NTCP transgenic mice is biphasic

**DOI:** 10.1128/mbio.01008-23

**Published:** 2023-07-12

**Authors:** Stephanie Maya, Leeor Hershkovich, E. Fabian Cardozo-Ojeda, Elham Shirvani-Dastgerdi, Jay Srinivas, Louis Shekhtman, Susan L. Uprichard, Andrew R. Berneshawi, Thomas R. Cafiero, Harel Dahari, Alexander Ploss

**Affiliations:** 1 Department of Molecular Biology, Princeton University, Princeton, New Jersey, USA; 2 Department of Medicine, The Program for Experimental & Theoretical Modeling, Division of Hepatology, Stritch School of Medicine, Loyola University Chicago, Maywood, Illinois, USA; 3 Vaccine and Infectious Disease Division, Fred Hutchinson Cancer Research Center, Seattle, Washington, USA; Virginia Polytechnic Institute and State University, Blacksburg, Virginia, USA

**Keywords:** viral hepatitis, hepatitis delta virus, hepatitis B virus, mathematical modeling, animal model

## Abstract

Chronic infection with hepatitis B and delta viruses (HDV) is the most serious form of viral hepatitis due to more severe manifestations of an accelerated progression to liver fibrosis, cirrhosis, and hepatocellular carcinoma. We characterized early HDV kinetics post-inoculation and incorporated mathematical modeling to provide insights into host-HDV dynamics. We analyzed HDV RNA serum viremia in 192 immunocompetent (C57BL/6) and immunodeficient (NRG) mice that did or did not transgenically express the HDV receptor—human sodium taurocholate co-transporting polypeptide (hNTCP). Kinetic analysis indicates an unanticipated biphasic decline consisting of a sharp first-phase and slower second-phase decline regardless of immunocompetence. HDV decline after re-inoculation again followed a biphasic decline; however, a steeper second-phase HDV decline was observed in NRG-hNTCP mice compared to NRG mice. HDV-entry inhibitor bulevirtide administration and HDV re-inoculation indicated that viral entry and receptor saturation are not major contributors to clearance, respectively. The biphasic kinetics can be mathematically modeled by assuming the existence of a non-specific-binding compartment with a constant on/off-rate and the steeper second-phase decline by a loss of bound virus that cannot be returned as free virus to circulation. The model predicts that free HDV is cleared with a half-life of 35 minutes (standard error, SE: 6.3), binds to non-specific cells with a rate of 0.05 per hour (SE: 0.01), and returns as free virus with a rate of 0.11 per hour (SE: 0.02). Characterizing early HDV-host kinetics elucidates how quickly HDV is either cleared or bound depending on the immunological background and hNTCP presence.

**IMPORTANCE:**

The persistence phase of HDV infection has been studied in some animal models; however, the early kinetics of HDV *in vivo* is incompletely understood. In this study, we characterize an unexpectedly HDV biphasic decline post-inoculation in immunocompetent and immunodeficient mouse models and use mathematical modeling to provide insights into HDV-host dynamics.

## INTRODUCTION

Hepatitis delta virus (HDV) is a single-stranded RNA virus belonging to the *Kolmioviridae* family, genus Deltavirus that requires the hepatitis B virus (HBV) envelope proteins to package infectious virions and spread (1, 2). While it has been previously estimated that approximately 15 million people are chronically infected with HDV, a recent meta-analysis of epidemiological studies published between 1977 and 2016 suggests that this number may be closer to 60–70 million (3). This estimation revolves around the current understanding that HDV solely exploits HBV envelope proteins (4 - 6); however, recent data argue the possibility that HDV might utilize envelope proteins of a variety of viruses (7 - 10), although its implication in natural settings is still unknown (11 - 13). There is firm clinical data that suggests that co-infection with HDV and HBV results in significantly worse and accelerated progression to liver fibrosis, cirrhosis, and hepatocellular carcinoma than HBV infection alone (14, 15). Nevertheless, by utilizing HBV surface antigens (HBsAg) as its envelope proteins, HDV infects hepatocytes by first attaching to heparan sulfate proteoglycans (HSPGs) and subsequently binding to the HBV receptor, human sodium taurocholate co-transporting polypeptide (hNTCP) (5, 16, 17). Differences in the sequence between human NTCP and non-permissive species explain in part the limited host tropism of HBV and by extension HDV. While HDV, mediated through the preS1 region of the HBsAg, can bind to mouse NTCP, differences in the amino acids 84–87 of the murine ortholog prevent HBV glycoprotein-mediated uptake (18). Expression of human NTCP is sufficient to mediate HDV uptake and infection in mouse hepatocytes *in vitro* (19), but these cells remain resistant to HBV (20, 21). These observations were corroborated in mice transgenically expressing hNTCP (22, 23) or a humanized allele of NTCP (18) that supports HDV. However, susceptibility was age-dependent and required inoculation with very high doses of HDV. We have subsequently shown that hNTCP transgenic (tg) mice support hepadnavirus glycoprotein-mediated uptake and persistent HDV infection when HBsAg is co-expressed (23).

The hNTCP tg mouse model allows us to investigate the kinetics of early HDV infection in mice. HDV long-term kinetics has been delineated in a few studies in humanized mice (23 - 26). Yet, HDV dynamics early during the infection, in particular the rate by which the virus enters hepatocytes or is cleared by the host, depending on host background immunity, remains to be defined.

In this study, we analyzed and mathematically modeled early HDV kinetics in hNTCP and non-hNTCP mice on an immunocompetent or immunodeficient background from inoculation until HDV viremia reached undetectable levels [lower limit of quantification (LLoQ)]. To minimize the number of potentially confounding variables, we analyzed the kinetics of HDV injection in hNTCP mice in the absence of HBV or HBsAg. Although HDV requires a helper virus to propagate, evidence has shown that HDV can persist in human hepatocytes for at least 6 weeks (27) demonstrating that HBV is not needed for establishing intracellular HDV replication.

In the current study, we identified two distinct phases of viral decline, indicating a rapid initial virus decline followed by a slower decline phase until reaching LLoQ. Because of very frequent blood sampling, it was also possible to estimate the rate of HDV clearance from blood and explain the nature of the biphasic decline via mathematical modeling.

## MATERIALS AND METHODS

A detailed description of the Materials and Methods used in this study is included in the supplementary information.

## RESULTS

### HDV RNA in the serum of hNTCP transgenic and non-transgenic mice undergoes a biphasic decline after single HDV inoculation

**Fig 1:**
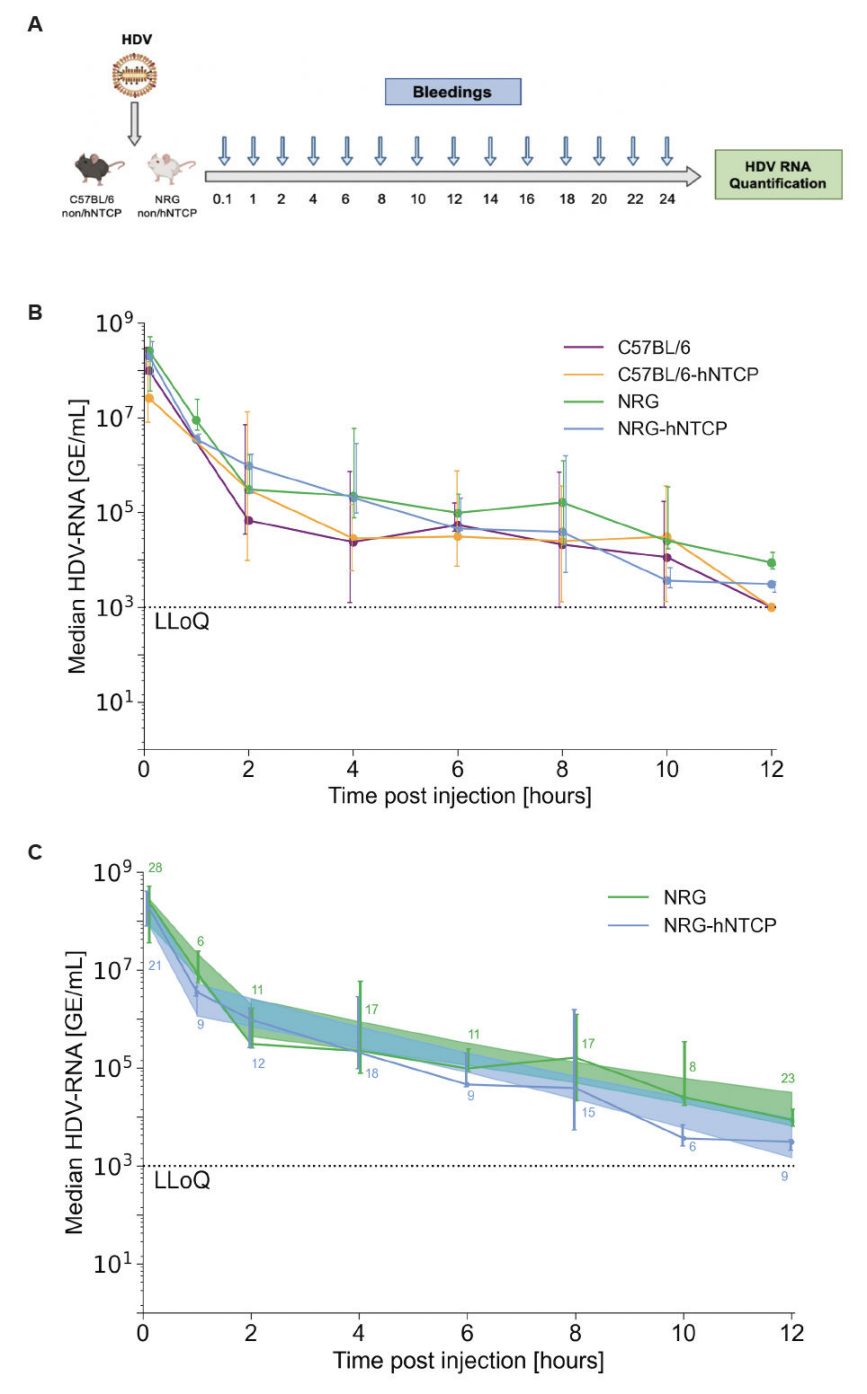
Single injection of HDV in C57BL/6-hNTCP or non-hNTCP mice and NRG-hNTCP or non-hNTCP mice. (**A**) Schematic of C57BL/6 and NRG non-hNTCP or hNTCP mice infected with HDV and bled every 2 hours for the first 24 hours. Viral RNA was quantified from the serum by RT-qPCR. The schematic was created with Biorender.com. (**B**) Serum HDV RNA quantification over the first 12 hours of infection in C57BL/6 and NRG non-hNTCP or hNTCP mice. (**C**) Median serum HDV RNA for all HDV-naive (first inoculation if applicable, including mice that were re-inoculated at 4 and 12 hours, but their data are cut off before second inoculation) NRG and NRG-hNTCP mice, along with linear regressions and shaded 95% confidence intervals. LLoQ indicates the lower limit of quantification (1,000 GE/mL). The second-phase decline is significantly steeper in NRG-hNTCP mice than in NRG mice [*P* = 0.05, analysis of covariance (ANCOVA) test]. All data points are represented as medians, with error bars representing interquartile range (IQR). The number of mice summarized by each point for NRG mice is shown in (**C**) green above the lines, and the number for NRG-hNTCP mice is shown in blue below the lines. Each timepoint for both C57BL/6 and C57BL/6-hNTCP represents the median of six mice, except for hour 12 which represents three mice.

To characterize the early kinetics of HDV in mice, we first sought to delineate the stability of HDV in mouse serum after injection in the absence of HBV or ongoing production of HBV surface antigen. To this end, we utilized hNTCP transgenic or non-transgenic mice either on the C57BL/6 or NRG (*NOD Rag1^-/-^ IL2Rg^NULL^ NOD.Cg-Rag1^tm1Mom^ Il2rg^tm1Wjl/SzJ^*) background, the latter lacking functional B, T, and natural killer (NK) cells. These cohorts of mice were intravenously administered 1 × 10^9^ genomic equivalents (GEs) of cell-culture-produced HDV and bled every 2 hours for 24 hours (Fig. 1A). Viral RNA was extracted from mouse serum and analyzed by RT-qPCR (quantitative reverse transcription PCR) at each timepoint.

HDV RNA levels in the singly inoculated C57BL/6-hNTCP (*n* = 18) and C57BL/6 (*n* = 18) mice at 1 minute post-injection (mpi) were not significantly different between the two experimental groups (Fig. 1B). Thereafter, HDV RNA levels in the serum of C57BL/6-hNTCP mice followed a rapid decline within the first 4 hours post-injection (hpi) followed by a slower decrease of serum HDV RNA which fell under the LLoQ by 12 hpi. In C57BL/6 mice, HDV viremia also declined swiftly in the first 4 hours followed by a decrease in LLoQ. Therefore, both C57BL/6 and C57BL/6-hNTCP mice followed a similar biphasic kinetic pattern characterized by a sharp first-phase decline and a slower second-phase clearance.

HDV RNA copy numbers in the inoculated NRG (*n* = 30) and NRG-hNTCP (*n* = 20) mice followed a similar biphasic decline within the first 4 hpi as their immunocompetent counterparts (Fig. 1B). This rapid drop in HDV RNA levels was likewise followed by a slower decrease from 4 to 12 hpi, after which viremia plateaued at very low levels in both groups. Specifically, we observed that HDV RNA levels in NRG-hNTCP mice decreased more rapidly than in NRG mice in the first hour post-injection, which was then followed by a slower decline (Fig. 1C). Overall, however, they both reached the LLoQ by 12 hpi.

Altogether, a biphasic viral decline was observed beginning with a sharp decrease in viral load at 4 hpi in all mouse cohorts, followed by a slower second decline phase. Viremia in the immunocompetent mouse cohorts fell near the LLoQ by 12 hpi, while viremia of the immunodeficient mice reached the LLoQ by 12 hpi.

### Viral entry inhibition by bulevirtide treatment in NRG-hNTCP mice had negligible effect on viral clearance

**Fig 2:**
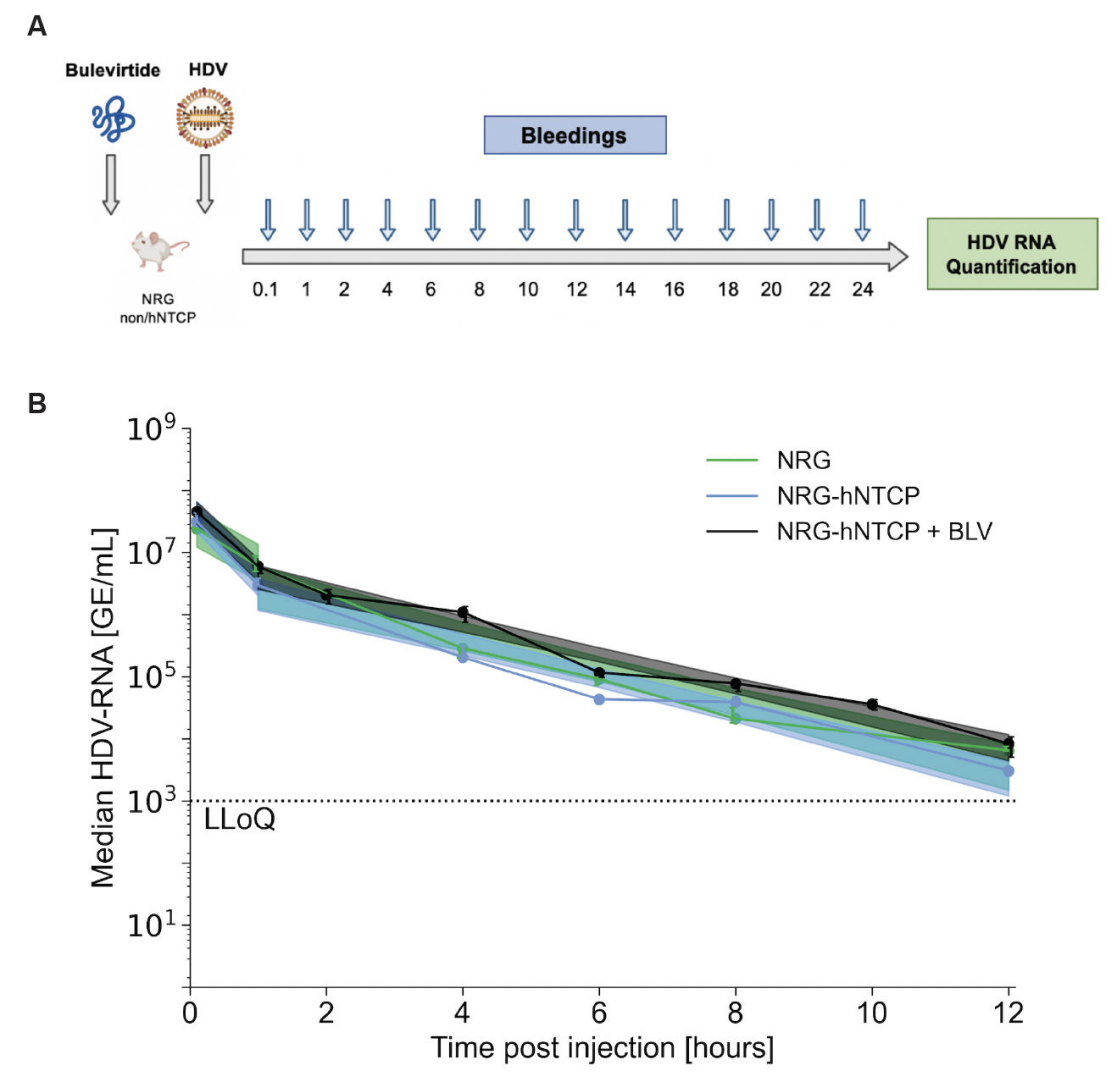
Bulevirtide (BLV) treatment 1 hour prior to single injection of HDV in NRG-hNTCP or non-hNTCP mice. (**A**) Schematic of NRG non-hNTCP or hNTCP mice pre-treated with bulevirtide and subsequently injected with HDV, followed by bleedings every 2 hours for the first 24 hours. Viral RNA was quantified from the serum by RT-qPCR. The schematic was created with Biorender.com. (**B**) Serum HDV RNA quantification over the first 12 hours of infection in HDV-infected NRG, NRG-hNTCP, or bulevirtide-treated NRG-hNTCP (NRG-hNTCP + BLV) mice. Linear regression confidences are displayed as shaded 95% confidence intervals. LLoQ indicates the lower limit of quantification (1,000 GE/mL). All data points are represented as medians, with error bars representing IQR. Each timepoint for each of the NRG, NRG-hNTCP, and NRG-hNTCP + BLV series is the median of three mice.

Since hepatocytes of NRG-hNTCP mice are permissive to HDV (23), we sought to determine whether the decline of the virus in the blood circulation is due to viral binding to hNTCP. We employed treatment with bulevirtide (also known as Myrcludex B or Hepcludex), an HBV/HDV entry inhibitor (23, 28, 29), to block viral binding to hNTCP by competitive inhibition. NRG-hNTCP mice (*n* = 9) were treated with bulevirtide 1 hour prior to inoculation with 1 × 10^8^ GE of HDV (Fig. 2A). Untreated NRG (*n* = 6) and NRG-hNTCP (*n* = 6) mice were also injected with 1 × 10^8^ GE HDV per mouse and were bled immediately after inoculation followed by bleedings every 2 hours for 24 hpi. Viral HDV RNA was extracted from mouse serum and analyzed by RT-qPCR at each timepoint.

In NRG-hNTCP mice treated with bulevirtide (bulevirtide-hNTCP), HDV RNA levels at 1 mpi were not significantly different between NRG-hNTCP and bulevirtide-hNTCP groups (Fig. 2B). Thereafter, serum HDV levels in NRG-hNTCP mice followed a biphasic decline characterized by a rapid drop from 0 to 2 hpi [1.1 log/hour (95% confidence interval, 95% CI: 0.77–1.5)] and a slower decrease from 2 to 12 hpi [0.27 log/hour (95% CI: 0.23–0.31)]. The biphasic decline kinetics in bulevirtide-hNTCP mice similarly consisted of a rapid phase decline in the first 2 hours [1.0 log/hour (95% CI: 0.74–1.3)] and a slower phase until 12 hpi [0.25 log/hour (95% CI: 0.22–0.28)] (Fig. 2B). HDV RNA levels in NRG-hNTCP mice pre-treated with bulevirtide did display a small but detectable delay in clearance in the serum compared to untreated NRG-hNTCP mice (Fig. S2). This could potentially be a result of a block in HDV uptake into hepatocytes due to bulevirtide competition; however, this observation is not statistically significant. The similar kinetics in mice on an NRG-hNTCP background, with or without bulevirtide, suggest the negligible effect of HDV binding on the decrease of virus in the bloodstream.

### Re-inoculation of HDV at 4 hpi in NRG-hNTCP transgenic and non-transgenic mice results in similar biphasic declines

**Fig 3:**
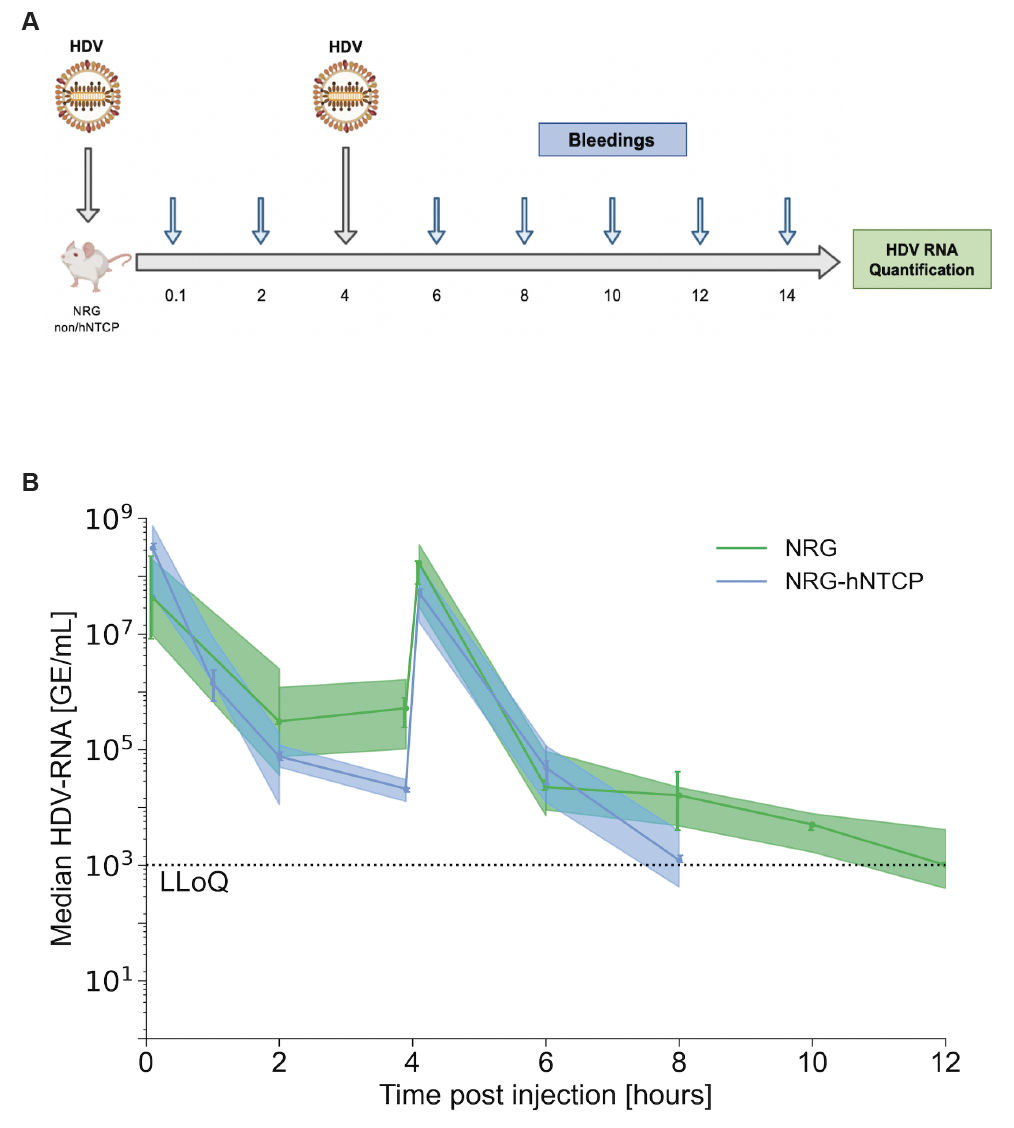
Re-injection of HDV in NRG-hNTCP mice 4 hours post-initial infection. (**A**) Schematic of NRG and NRG-hNTCP mice injected with HDV at time zero and 4 hpi and bled every 2 hours for the first 24 hours. Viral RNA was quantified from the serum by RT-qPCR. The schematic was created with Biorender.com. (**B**) Median serum HDV RNA quantification over the first 12 hours of infection in NRG and NRG-hNTCP mice, along with linear regressions and shaded 95% confidence intervals. LLoQ indicates the lower limit of quantification (1,000 GE/mL). All data points are represented as medians, with error bars representing IQR. Each timepoint in both the NRG and NRG-hNTCP time series represents the median value of three mice.

To further investigate whether receptor saturation or another binding site is the cause of this biphasic decline, we injected mice with HDV a second time at 4 or 12 hours following the initial inoculation, which characterized the end of each kinetic phase in single inoculation experiments. To observe the effect of re-inoculation at the end of the first phase, we utilized NRG (*n* = 18) and NRG-hNTCP (*n* = 15) mice and injected them with HDV at 1 × 10^8^ GE per mouse followed by re-inoculation at 4 hpi with the same viral load (Fig. 3A).

In doubly injected NRG and NRG-hNTCP mice, the HDV RNA levels followed a biphasic decline in accordance with a rapid drop from 0 to 2 hpi and a slower decrease from 2 to 4 hpi (Fig. 3B). After re-injection, HDV RNA levels reached a peak around 1 × 10^8^ GE/mL, which did not differ significantly from the initial RNA levels following the first re-injection. Afterward, RNA levels followed a biphasic decline consistent with an initial rapid drop from initial levels in NRG mice and NRG-hNTCP mice. Notably, while the early rapid phase lasted 2 hours (from 4 to 6 hpi) post-re-injection in NRG mice, the rapid phase in NRG-hNTCP mice lasted 4 hours post-re-injection, from 4 to 8 hpi (Fig. 3B). The rapid decline was then followed by a slower phase decrease in both mouse cohorts. Strikingly, levels of HDV RNA in the NRG-hNTCP mice decreased more rapidly after both HDV injections as compared to NRG mice. The second injection even resulted in the clearance of viral RNA in the serum of NRG-hNTCP mice by 8 hpi while NRG mice experienced viral RNA clearance by 12 hpi.

### Re-inoculation of HDV at 12 hpi in NRG-hNTCP transgenic and non-transgenic mice results in slower second-phase declines compared to 4 hpi re-inoculation

**Fig 4:**
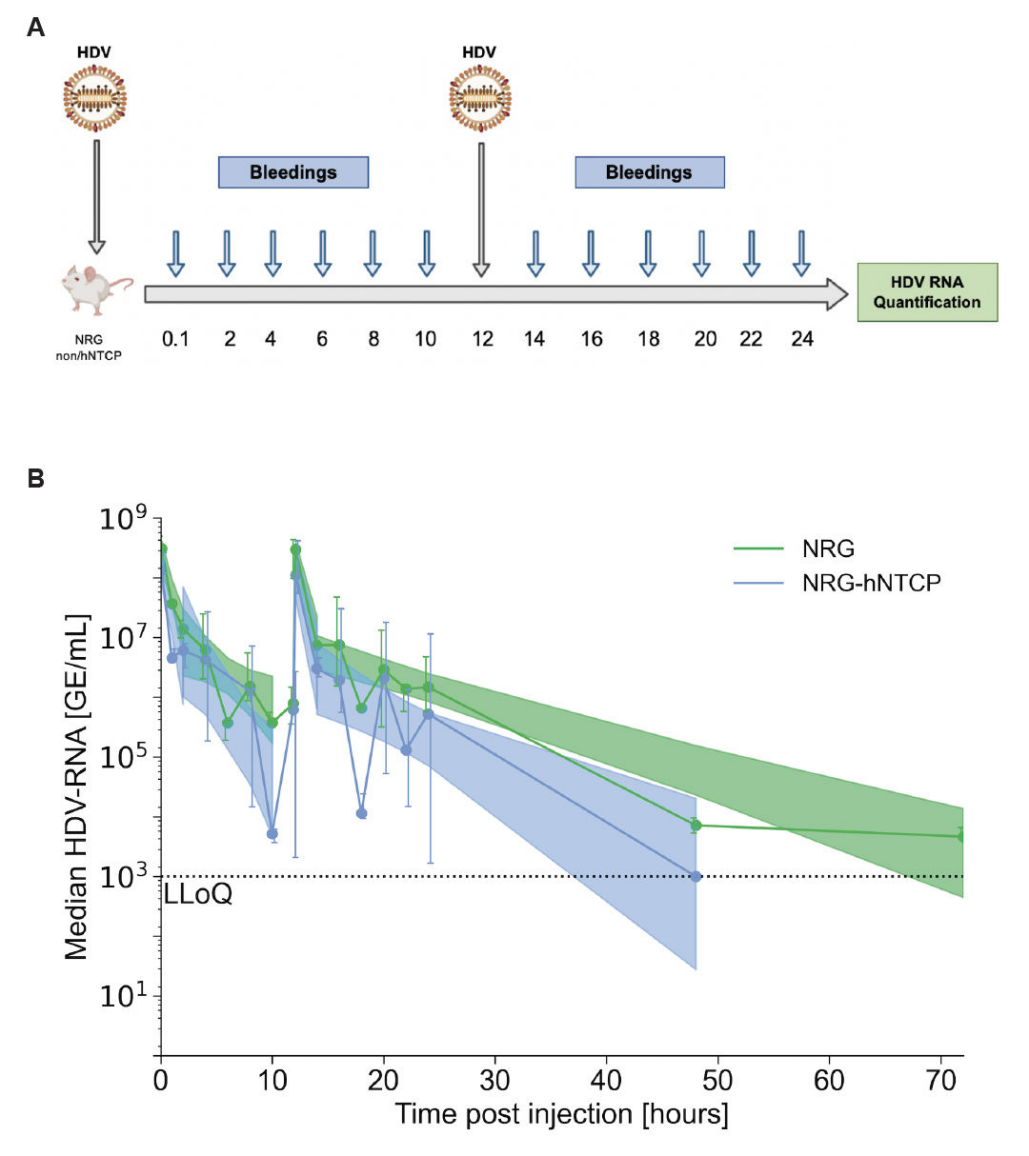
Re-injection of HDV in NRG and NRG-hNTCP mice 12 hours post-initial infection. (**A**) Schematic of NRG and NRG-hNTCP mice injected with HDV at time zero and 12 hpi and bled every 2 hours for the first 24 hours. Viral RNA was quantified from the serum by RT-qPCR. A schematic was created with Biorender.com. (**B**) Serum HDV RNA quantification over all 72 hours of infection in NRG and NRG-hNTCP mice and linear regressions with shaded 95% confidence intervals. LLoQ indicates the lower limit of quantification (1,000 GE/mL). The second-phase decline after the second inoculation is significantly steeper in NRG-hNTCP mice than in NRG mice (*P* = 0.02, ANCOVA test). Slope comparisons for the first inoculation are presented in Fig. 1. All data points are represented as medians, with error bars representing IQR. Each timepoint is representative of three mice.

To probe the potential for receptor and/or other binding site saturation during the second-phase decline of HDV, we re-inoculated the mice at 12 hpi. Thereby, we reasoned that we can determine whether there is a sharper or steadier decline in viral RNA when HDV is re-introduced during the second phase of decline. NRG (*n* = 12) and NRG-hNTCP (*n* = 15) mice were thus injected at 0 hpi and 12 hpi with bleedings every 2 hours for the first 24 hours (Fig. 4A).

In both cohorts, HDV RNA biphasic decline comprised a rapid drop from 0 to 4 hpi and a slower phase decline from 4 to 12 hpi (Fig. 4B). The biphasic decline kinetics in both groups consisted of a rapid phase decline and a slower phase decline until 12 hpi, after which the mice were re-injected with a second HDV inoculation dose. After re-injection, median HDV RNA levels reached a peak that was not significantly different from the RNA levels at the initial injections. Subsequently, HDV RNA levels followed a biphasic decline composed of an initial drop in RNA levels followed by a slower phase decrease. Remarkably, the HDV levels in the NRG mice did not fall below the LLoQ by 48 hpi as in the NRG-hNTCP mice; instead, the levels remained elevated until 72 hpi (Fig. 4B). Similar to the 4 hpi injections in Fig. 3B, the NRG-hNTCP mice resulted in faster declines of HDV RNA in the serum following both injections compared to NRG mice. To analyze the potential impact of early immune responses, we also conducted the 12 hpi re-inoculation in transgenic and non-transgenic C57BL/6 mice. Comparatively, RNA levels in C57BL/6 and C57BL/6-hNTCP mice followed similar kinetics to NRG and NRG-hNTCP mice (Fig. S1A and B).

Overall, a biphasic decline before/after re-inoculation was observed in both 4 hpi and 12 hpi cohorts for transgenic and non-transgenic NRG mice (Fig. 3B and 4B). Particularly, HDV RNA levels in NRG-hNTCP mice declined more rapidly following double HDV injections as compared to NRG mice. These data suggest that the hNTCP receptors for HDV binding are not saturated during early infection.

### Agglomerate kinetic analysis reveals faster clearance of NRG-hNTCP mice compared to NRG mice after re-inoculations

To assess the overall effects and trends of the hNTCP receptor, immunocompetence, and of bulevirtide on HDV viral kinetics, mice were agglomerated for further analysis across experiment runs and cohorts.

On the first intravenous injection of HDV in HDV-naive mice (data from re-inoculated mice were cut off at the time of re-inoculation), all mouse strains followed a similar biphasic decline (Fig. 1B). HDV RNA rose to a median of 8.3 log GE/mL (interquartile range, IQR 7.6–8.6). NRG mice experienced a rapid initial median decline in HDV RNA of 1.2 log/hour until 2 hpi, followed by a slower median decline of 0.19 log/hour (95% CI: 0.13–0.25 log GE/mL) until 12 hpi. NRG-hNTCP mice experienced a similar rapid initial median decline in HDV RNA of 1.9 log/hour until 1 hpi, followed by a slower second median decline of 0.26 (95% CI: 0.20–0.31) log/hour (Fig. 1C; Table 1).

**TABLE 1:** Summary of first inoculation agglomerate mouse kinetics for all mice, excluding mice that were administered bulevirtide^*a*^

	NRG	NRG-hNTCP	C57BL/6	C57BL/6-hNTCP
*N* (male, female)	66 (24, 24)^*b*^	53 (27, 26)	25 (0, 6)^*b*^	22 (15, 7)
Mean baseline HDV RNA (log GE/mL) after inoculation (95% CI)	8.21 (6.90, 9.16)	8.19 (7.10, 8.97)	7.81 (5.99, 8.64)	7.60 (6.87, 8.68)
First-phase viral decline slope (log GE/mL/hour) (95% CI)	1.2 (0.95, 1.5)	1.9 (1.5, 2.4)	1.2 (0.28, 2.0)	1.1 (1.2, 2.0)
Second-phase viral decline slope (log GE/mL/hour) (95% CI)	0.19^*c*^ (0.13, 0.25)	0.26^*c*^ (0.20, 0.31)	0.19 (0.05, 0.33)	0.17 (0.03, 0.31)

^*a*^ Data represented include all HDV-naïve mice, including those that only received one inoculation and those that were re-inoculated (including at 4 and 12 hours), but their data are cut off before the second inoculation. Each mouse strain studied is represented as a single agglomerate distribution of all re-inoculation time groups. The 95% confidence intervals (CIs) for each decline are shown, demonstrating the steeper second-phase decline in NRG-hNTCP mice.

^*b*^ Numbers in parentheses represent the known sex of mice within this subpopulation, and the remainder are unspecified.

^*c*^ These slopes are significantly different from each other (*P* = 0.05).

The HDV RNA kinetics of C57BL/6 and C57BL/6-hNTCP mice did not differ significantly between each other or between their immunocompromised counterparts (NRG/NRG-hNTCP) (Fig. 1B). Similarly, the administration of bulevirtide to NRG-hNTCP mice did not affect the slope of decline compared to untreated NRG and NRG-hNTCP mice in the same cohort (Fig. 2B; Table 1). Due to their negligible effects and their smaller sample sizes, C57BL/6 and C57BL/6-hNTCP mice were precluded from mathematical modeling.

Following the re-inoculations at 4 or 12 hpi of NRG and NRG-hNTCP, the median HDV RNA rose to 8.14 log GE/mL (IQR: 7.78–8.59 log GE/mL). Just as in the first inoculation, all mice showed a biphasic HDV RNA decline post re-inoculation. For both NRG and NRG-hNTCP mice, the viral kinetics of mice re-inoculated at 4 hpi differed from those re-inoculated at 12 hpi (Fig. 3B and 4B). The NRG mice re-inoculated at 4 hpi experienced a median rapid first-phase decline in HDV RNA of 1.9 (95% CI: 1.5–2.3) log/hour for 1 hour, followed by a slower decline of 0.23 (95% CI: 0.09–0.36) log/hour to LLoQ. The NRG mice that were re-inoculated at 12 hpi showed a comparatively slower rapid first phase of HDV RNA/decline of 0.79 (95% CI: 0.5–1.1) log/hour for 3 hours, followed by a slower second-phase decline of 0.06 (95% CI: 0.04–0.07) log/hour. The NRG-hNTCP mice that were re-inoculated at 4 hpi experienced a median rapid first-phase HDV RNA decline of 1.6 (95% CI: 1.3–2.0) log/hour for 1 hour, followed by a slower decline of 0.73 (95% CI: 0.39–1.1) log/hour. The NRG-hNTCP mice that were re-inoculated at 12 hpi had a median rapid first-phase HDV RNA decline of 0.82 (95% CI: 0.39–1.2) log/hour for 3 hours, followed by a slower second phase of 0.10 (95% CI: 0.05–0.15) log/hour (Table 2).

**TABLE 2:** Summary of NRG and NRG-hNTCP second inoculation agglomerate kinetics^*a*^

	NRG		NRG-hNTCP	
Re-inoculation time	4	12	4	12
*N* (male, female)	18^*b*^	12 (10, 2)	13 (7, 6)	12 (4, 8)
Mean baseline HDV RNA (log GE/mL) after inoculation (95% CI)	8.01 (7.54, 8.29)	8.37 (7.89, 8.70)	7.70 (7.55, 7.84)	8.06 (7.23, 8.77)
First-phase viral decline slope (log GE/mL/hour) (95% CI)	1.9 (1.5, 2.3)	0.79 (0.50, 1.1)	1.6 (1.3, 2.0)	0.82 (0.39, 1.2)
Second-phase viral decline slope (log GE/mL/hour) (95% CI)	0.23 (0.09, 0.36)	0.06^*c*^ (0.04, 0.07)	0.73 (0.39, 1.1)	0.10*^c^* (0.05, 0.15)

^*a*^ For both NRG and NRG-hNTCP, the 4-hour re-inoculation and 12-hour re-inoculation groups are represented separately. The 95% confidence intervals for each decline are shown, demonstrating the steeper second-phase decline in NRG-hNTCP mice.

^*b*^ Numbers in parentheses represent the known sex of mice within this subpopulation, and the remainder are unspecified.

^*c*^ Significantly different slopes from each other (*P* = 0.002).

A steeper second-phase decline was observed in NRG-hNTCP mice relative to NRG, both after the first inoculation (*P* = 0.05; Fig. 1C) and after the second inoculation (*P* = 0.002; Fig. 4B). The consistent steeper second-phase decline suggests that there may be a heightened immune response or receptor-bound virus in the second-phase decline for NRG-hNTCP mice. To provide insights into HDV-host kinetics in the NRG and NRG-hNTCP mice, we developed a mathematical model as described below.

### A binding compartment mathematical model can be used to explain the biphasic viral decline in mice

**Fig 5:**
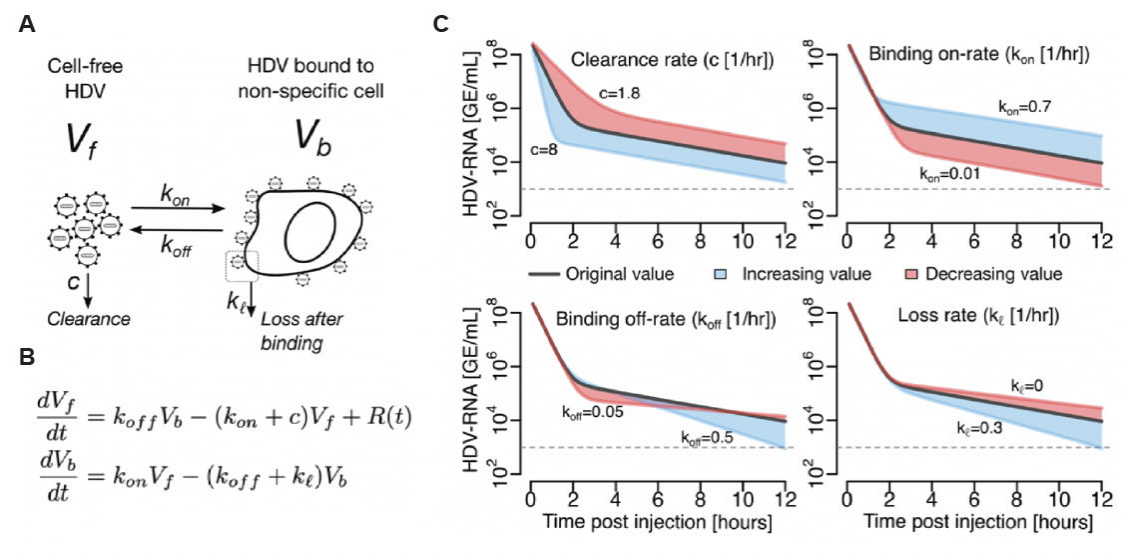
Binding compartment mathematical modeling. (**A**) Schematic of mathematical model: $V_{f}$ is the free-roaming virions and $V_{b}$ is the cell-bound HDV. Free virus enters circulation by being released from a non-specific binding cell with rate constant $k_{off}$ and is removed from circulation at a general clearance rate c , as well as by being bound to a cell at rate constant $k_{on}$ . The cell-bound $V_{b}$ shrinks as it is released from the binding cell at rate constant $k_{off}$ or as it is lost with rate $k_{l}$ only in the transgenic NRG-hNTCP mice. (**B**) Model equation. Here, $R\left(t\right)$ is a pulse function with the form $R\left(t\right)=\left\{ \begin{array}{ll} 10^{r}, & t_{in}<t<t_{in}+0.1\\ 0, & otherwise \end{array}\right.$ , with $t_{in}$ the time of inoculation or re-inoculation of HDV (0, 4, or/and 12 hours) and r the orders of magnitude by which cell-free HDV increases during the inoculation or re-inoculation of the virus. (**C**) Model simulations indicate that the speed and time of the first phase of decline are primarily affected by the HDV clearance rate (top-left) and the binding on-rate (top-right). The second phase of decline is primarily affected by the binding off-rate (bottom-left) and internalization rate parameters (bottom-right). Model parameters in each graph are fixed at: $k_{on}$ = 0.07 per day, $k_{off}$ = 0.22 per day, $k_{l}$ = 0.1 per day, c = 3.71 per day (black curve). Adjustments made to each parameter are shown in red (underestimate) and blue (overestimate), for illustrative purposes.

Assuming no viral production and negligible effect of HDV entry on viral decline, a binding compartment model (Fig. 5A and B) was built to describe the experimental observations from the NRG and NRG-hNTCP mice. The model considers the dynamics of two populations of virus, the free-roaming virions, *V*_*f*_ , and cell-bound HDV, *V*_*b*_ . Assuming no production of new virions, free virus enters circulation by being released from a bound cell with rate constant and is removed from circulation at a general clearance rate , as well as by being bound to a cell at rate constant . The cell-bound population grows as is bound to a cell with rate constant and shrinks as is released from the binding cell at rate constant or as it is lost (with no unbinding) with rate only in the transgenic NRG-hNTCP mice (Fig. 5A). To account for the injection or re-injection of HDV at times 0, 4, or 12 hours, the function was incorporated (Fig. 5B). Here, is a pulse function with the form , with the time of inoculation or re-inoculation of HDV (0, 4, or/and 12 hours) and the orders of magnitude by which cell-free HDV increases during inoculation or re-inoculation of virus.

To examine the model (Fig. 5A and B) sensitivity to model parameters, a one-way sensitivity analysis was conducted (Fig. 5C). The cell-free virus ( $V_{f}$ ) clearance rate constant ( c ) was positively associated with the slope and duration of the first phase of $V_{f}$ decline, and the binding rate constant ( $k_{on}$ ) was inversely correlated with the duration of the first phase of HDV decline. The second phase of $V_{f}$ decline is primarily associated with the loss and off-rate constant after binding (i.e., a larger binding compartment internalization and off-rate are associated with a faster second-phase decline rate). The loss and off-rate constant ( $k_{l}$ and $k_{off}$ , respectively) were positively associated with the slope of the second-phase decline. Similarly, simulations predict that the majority of $V_{f}$ rapidly become cell-bound ( $V_{b}$ ), which reach peak values followed by $V_{b}$ decline (Fig. S2). The peak in $V_{b}$ is inversely correlated with changes in clearance rate ( c ) and positively correlated with the binding rate constant ( $k_{on}$ ). The second-phase decline rate was governed by the loss and off-rate constant ( $k_{l}$ and $k_{off}$ , respectively), where a larger binding compartment loss and off-rate are associated with a faster second-phase decline.

### Modeling suggests NRG-hNTCP mice experience a loss of bound HDV that does not return to circulation as free virus

**Fig 6:**
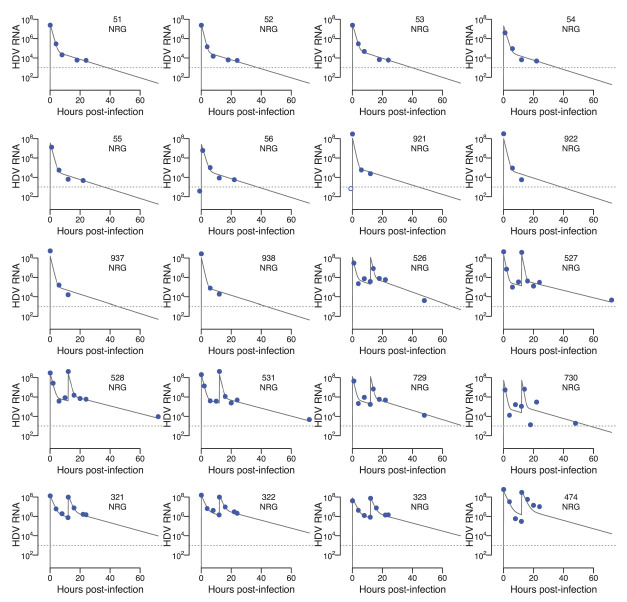
Best mathematical model fits of NRG mice using the maximum likelihood estimates (MLEs) of the population parameter distributions. Best model fits for some of the HDV concentrations (GE/mL) from the NRG mice in the presence or absence of HDV re-inoculation at 12 hours after infection. Dotted horizontal line is the lower limit of quantification (LLoQ). Dark blue circles are data from NRG mice, filled circles are data above LLoQ and empty circles are data below the LLoQ, respectively. Individual parameter estimates are given in Table. S1. See Materials and Methods in the supplementary information for details of the assumptions for population distributions.

**Fig 7:**
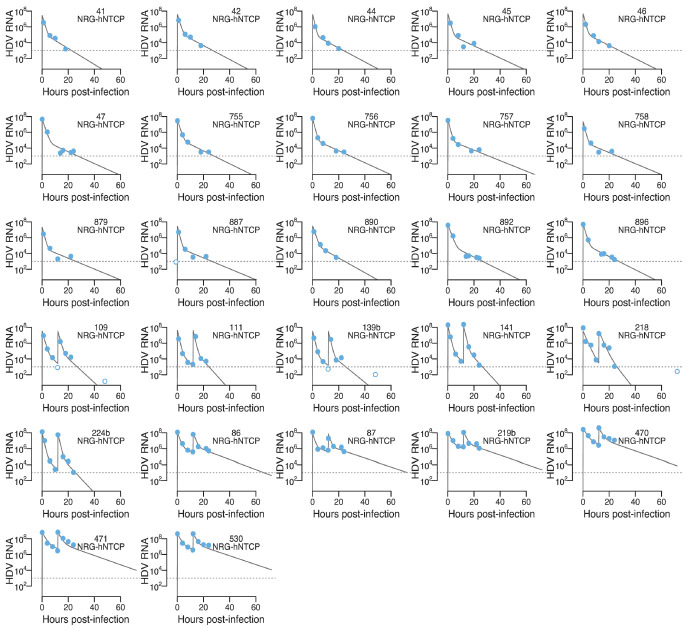
Best mathematical model fits of NRG-hNTCP mice using the maximum likelihood estimates (MLEs) of the population parameter distributions. Best model fits for the HDV concentrations (GE/mL) from NRG-hNTCP mice in the presence or absence of HDV re-inoculation at 12 hours after infection. Dotted horizontal line is the lower limit of quantification (LLoQ). Light blue circles are data from NRG-hNTCP mice, filled circles are data above LLoQ and empty circles are data below the LLoQ, respectively. Individual parameter estimates are in Table S2. See Materials and Methods in the supplementary information for details of the assumptions for population distributions.

We simultaneously fit this model to the NRG and NRG-hNTCP mouse data using a non-linear mixed effect approach. We excluded mice without more than one data point for each viral decline phase for the fits. We tested a model with a covariate for $k_{l}$ (i.e., k_l_ > 0 for the NRG-hNTCP group), representing that bound virus in NRG-hNTCP mice gets lost leading to a faster second phase. We compared this model with respect to a null model, where $k_{l}=0$ (without a covariate for $k_{l}$ ). Using model selection, we found that a model assuming k_l_ > 0 is more parsimonious to explain the data [ΔAICc (Akaike Information Criteria) = 11.1]. Model fits with measured viral load from all (NRG and NRG-hNTCP) mice with sufficient frequent data are shown in Fig. 6 and 7, respectively, using the individual parameter estimates given in Table S1 and S2. The maximum likelihood estimates of the population distributions are shown in Table 3 (for more details on the model parameter definitions, see Materials and Methods section in the supplementary information).

**TABLE 3:** Mathematical model parameter estimates for the NRG and NRG-hNTCP mice population^*a*^

Parameter (units)	Description	Population estimate (SE)	Inter-individual variability (%RSE)
$V_{f}\;\left(0\right)$ (log_10_ GE/mL)	Baseline of HDV RNA	7.9 (0.10)	0.06 (17)
$k_{on}$ (1/hour)	Binding rate	0.05 (0.01)	0.99 (25)
$k_{off}$ (1/hour)	Dissociation rate	0.11 (0.02)	0.23 (41)
c (1/hour)	Clearance rate	1.18 (0.11)	0.44 (20)
$k_{l}$ (1/hour)	Loss rate after binding (in NRG-hNTCP mice)	0.07 (0.04) *P* < 0.001, Wald test	0.07 (30)

^*a*^ As explained in the text, the parameter value for *k_l_* only applies only for NRG-hNTCP mice (i.e., *k*_*l*_ = 0 for NRG mice). The table shows the maximum likelihood estimate (MLE) values for the fixed effects (population values) and the SD of the random effects (inter-individual variation). Wald statistical test evaluates that the covariate for *k*_*l*_ in the NRG-hNTCP group is greater than zero. SE: standard error; %RSE: % relative SE.

As presented in the previous section, this model can recapitulate the biphasic decline in HDV concentration from all mice and can interpret the faster decline in the second phase for the NRG-hNTCP mice. From the best model fits, we found that free HDV is cleared with a rate 1.2 per hour (standard error, SE = 0.11) equivalent to a half-life of 35 minutes (SE = 6.3), binds to non-specific cells with rate of 0.05 per hour (SE = 0.01), and returned as free virus with rate of 0.11 per hour (SE = 0.02) (Table 3). The best model also shows that $k_{l}$ in the NRG-hNTCP mice is significantly greater than zero (*P* < 0.001, Wald test) with a rate of 0.07 per hour (SE = 0.04). This result implies that NRG-hNTCP mice have a loss of HDV bound to non-specific cells that do not become free virus.

## DISCUSSION

Long-term HDV infection kinetics has been previously characterized (23 - 25), but kinetics of HDV RNA levels early on post-inoculation has not been elucidated. The understanding of how quickly HDV RNA can either be cleared from the serum or attach/internalize into hepatocytes can aid the development and administration of anti-viral drugs to be more potent depending on these kinetics. In this study, we utilized immunodeficient mice that we previously established (23) to characterize early HDV kinetics in immunocompetent versus immunodeficient backgrounds. These immunodeficient mice are non-obese diabetic recombinase activating gene 1 knockout (*Rag1^-/-^*) interleukin 2 receptor gamma chain deficient (*IL-2Rγ^NULL^*) (NRG) mice, which lack functional NK cells, B and T lymphocytes (30), and express hNTCP (23). While hNTCP-transgenic mice are useful for studying HDV/HBV infection, we know little about early HDV kinetics in these mouse models. Therefore, we investigated the early kinetics of HDV infection in hNTCP transgenic mice on an immunocompetent (C57BL/6) or immunodeficient (NRG) background. We demonstrate that in all mice—irrespective of hNTCP expression and immune status—HDV RNA kinetics follows an unexcepted biphasic decline that is characterized by a sharp drop in the first phase and then a slower steady decrease until they reached LLoQ. Treating NRG-hNTCP mice with the HBV/HDV entry inhibitor, bulevirtide, suggests that viral entry is a minor contributor to HDV decline rates in NRG-hNTCP mice. Moreover, re-inoculating these mice with HDV 4 or 12 hpi still results in a biphasic decline of virus, suggesting that saturation of the interaction between HDV and its attachment factors—presumably HSPGs and/or hNTCP—is not achieved, which otherwise would have been expected to result in slowing viral clearance after the second inoculation. We employed roughly equal numbers of male (*n* = 92, 48%) and female (*n* = 100, 52%) mice and did not observe differences in HDV kinetics between the genders of mice.

To increase the likelihood that the HDV inoculation resulted in high serum viremia detectable within the limits of our RT-qPCR assay and to ensure that the majority of target cells would be exposed to the virus, we began our experiments using a large dose of 10^9^ GE/mouse followed by 10^8^ GE/mouse in subsequent experiments, as our laboratory has previously done (23). We note that throughout the study, we observed no phenotypical differences, such as size, bile acid levels, or clinical features, in the mice between wild-type and transgenic mice. Single inoculations of 10^9^ GE/mouse in immunocompetent (C57BL/6) and immunodeficient (NRG) mice either expressing hNTCP or not resulted in similar biphasic declines within the first 24 hours, with a sharp drop by 4 hpi followed by a slower decrease by 24 hpi. While the similar clearance rates were initially surprising, we reasoned that the adaptive immune response would not be primed within the first 24 hours of injection and therefore the initial HDV kinetics could resemble those in immunodeficient mice.

As the HDV RNA levels in NRG-hNTCP mice declined in a biphasic manner similarly to the other mouse cohorts, contrary to what was expected, we decided to analyze whether viral binding to NTCP was a factor to viral clearance in the blood through the use of bulevirtide, which binds to hNTCP and competitively inhibits HDV-receptor interactions. Since both hNTCP transgenic and non-transgenic C57BL/6 mouse cohorts exhibited viral decreases that were predicted due to their immunocompetency, we did not include them in this experiment. Bulevirtide treatment on HDV-injected NRG-hNTCP mice did not affect the slopes of viral decline compared to those of HDV-injected NRG or NRG-hNTCP mice (Fig. 2B), indicating that the hosts were able to eliminate the virus from circulation. This could be explained by bulevirtide binding non-specifically to HSPGs and/or binding to both endogenous mouse and human NTCP and thus resulting in similar kinetics of hNTCP and non-hNTCP mice. In this study, we were not able to detect HDV RNA over the background in the liver (data not shown). Prior work has established that HDV RNA is difficult to reliably quantify in the liver after mono-infections especially during early infection (22, 23), as HDV cannot spread and amplify without a helper virus; however, future experimentation should include a longitudinal study in which HDV RNA would be more readily available in liver tissue to determine whether non-specific binding or internalization decreased the levels of HDV RNA in the serum.

To conjecture as to why the HDV RNA levels followed the same pattern in all three of these cohorts during single injection and BLV treatment, we explored the possibility that NTCP was saturated by viral binding. We thus injected mice at the end of the first and second phases, which corresponded with the timepoints of 4 and 12 hpi, respectively. Two main putative scenarios arise here: either viral RNA remains steady over time whereby the viral load will not decrease or there is a biphasic decline, in which there is no HDV-binding receptor saturation. Following 4 hpi re-inoculations, HDV RNA levels in NRG-hNTCP fell under the LLoQ by 8 hpi, whereas RNA levels in NRG mice lasted over the LLoQ until 12 hpi. Moreover, HDV RNA levels in the 12 hpi re-inoculations of NRG-hNTCP mice fell under the LLoQ by 48 hpi, whereas RNA levels of NRG mice hovered over the LLoQ by 72 hpi, indicating that re-inoculation of HDV in NRG-hNTCP mice consistently resulted in a faster second phase compared to NRG mice. This is notable because single inoculation of HDV in these mice yielded similar biphasic declines in NRG-hNTCP mice as in NRG, C57BL/6, and C57BL/6 mice. We also found that immediately before re-inoculation, the viral RNA levels in the 4-hour re-inoculation (*P* = 0.1 for three NRG versus three NRG-hNTCP mice) and 12-hour re-inoculation (*P* = 0.88 for eight NRG versus nine NRG-hNTCP mice) were not significantly different. Moreover, re-inoculation of C57BL/6 and C57BL/6 -hNTCP mice emulated NRG kinetics, as the HDV RNA levels hovered above the LLoQ by 24 hpi (Fig. S1). HDV RNA levels in C57BL/6-hNTCP mice notably did not resemble those of NRG-hNTCP mice. We conjecture that this could be due to the clearance of the virus in the serum before the virus can attach to hNTCP. In contrast, in NRG-hNTCP mice, which are deficient in lytic complement and lack functional NK, B, and T cells, their immunocompromise status might delay viral clearance and thus allow binding of the virus to hNTCP. Thus, the steeper second-phase viral decline found in NRG-hNTCP mice compared to NRG mice suggests a role of hNTCP in the removal of virus from circulation.

We know that kinetics of the second-phase decline are not influenced by the production and release of virions in the bloodstream, as the mice do not produce HBV or HBsAg, nor should there be a significant amount of HDV in the blood occurring from HDV-injected hepatocyte death (23). Moreover, the hNTCP transgenic mice employed in this study were generated in our prior study (23), and RT-PCR anlaysis has demonstrated that hNTCP is solely expressed in liver tissue. We therefore developed a mathematical model that assumes free-roaming HDV virions in the blood that can be bound to cells non-specifically without productive infection or viral replication, reminiscent of the binding compartment model used in reference (31). The model (Fig. 5) is able to recapitulate the biphasic decline of the observed HDV concentration and suggests that: the first phase of viral decline is explained by viral clearance rate from blood (*c*) and binding on-rate ( $k_{on}$ ) of the free virus, and the second phase of decline by the dissociation of bound virus rate ( $k_{off}$ ) [partially mediated by interactions between the viral envelope between HSPG/hNTCP and/or apolipoproteins that may be associating with the virus—as shown for HBV (32)—and their respective receptors] plus its loss rate ( $k_{l}$ ) before dissociation only in hNTCP mice (either by disintegration or loss of bound virus that cannot return to circulation). Model fitting in NRG and NRG-hNTCP mice suggests that mice expressing the hNTCP receptor may sustain a significantly faster second phase of decline by virus loss after binding which cannot return to circulation (i.e., $k_{l}$ > 0 in the model for the NRG-hNTCP mice). We speculate that this can be explained by the virus entering hepatocytes and/or yet unexplained entry factor-virus interactions in transgenic hNTCP mice.

We estimated that the half-life of HDV in the bloodstream in our mouse model was 35 (SE = 6.3) minutes. Interestingly, the half-life of HBV in chimeric urokinase-type plasminogen activator/severe combined immunodeficiency mice was found to be ~twofold longer, i.e.,~1 hour (33). HBV is a DNA virus and consequently is more stable in the bloodstream, whereas HDV, being an RNA virus, is less stable and could theoretically be cleared more rapidly by the host due to the structural or nucleic acid degradation within the viral particles. Additionally, whether human hepatocytes clear HDV faster than mouse hepatocytes needs to be further investigated.

Elucidating HDV kinetics in these mouse models will further benefit from investigating several other aspects that were beyond the scope of this study. One such point is evaluating the effects of age on viral decline in these mouse models. Various studies have shown that young mice, those only 4 weeks of age or younger, are more susceptible to HDV infection and will develop chronic infections (18, 34). Additionally, including an HBsAg-producing mouse model will reveal whether HDV follows the same early kinetics or if newly synthesized virions could be found in the bloodstream 24 hpi. We did not inspect HDV replication in this study, but this would help illustrate the ability of HDV to enter the hepatocytes within the first 24 hours of infection or if the virus requires more time. Future studies can also build on the present study by evaluating bulevirtide treatment on HDV re-inoculations, providing insights into the nature of the observed biphasic HDV RNA decline in the bloodstream.

Altogether, this study demonstrates that adult C57BL/6, C57BL/6-hNTCP, NRG, and NRG-hNTCP mice undergo biphasic declines of viral RNA when inoculated with HDV in the absence of a helper virus, such as HBV. Remarkably, NRG-hNTCP mice displayed a more rapid second-phase decline compared to NRG mice following double inoculation. Future studies, including characterization of the innate immune cell response and theoretical analysis, will aid in the understanding of HDV-host dynamics in early infection.

## SUPPLEMENTAL MATERIAL

Supplemental information mbio.01008-23-s0001.pdf Supplemental figures, tables, and materials and methods.

Supplemental raw data mbio.01008-23-s0002.xlsx All raw data, including a figure legend.

ASM does not own the copyrights to Supplemental Material that may be linked to, or accessed through, an article. The authors have granted ASM a non-exclusive, world-wide license to publish the Supplemental Material files. Please contact the corresponding author directly for reuse.

## References

[1] Lempp FA , Schlund F , Rieble L , Nussbaum L , Link C , Zhang Z , Ni Y , Urban S . 2019. Recapitulation of HDV infection in a fully permissive hepatoma cell line allows efficient drug evaluation. Nat Commun 10:2265. doi:10.1038/s41467-019-10211-2 31118422 PMC6531471

[2] Abou-Jaoudé G , Sureau C . 2007. Entry of hepatitis delta virus requires the conserved cysteine residues of the hepatitis B virus envelope protein antigenic loop and is blocked by inhibitors of thiol-disulfide exchange. J Virol 81:13057–13066. doi:10.1128/JVI.01495-07 17898062 PMC2169099

[3] Chen HY , Shen DT , Ji DZ , Han PC , Zhang WM , Ma JF , Chen WS , Goyal H , Pan S , Xu HG . 2019. Prevalence and burden of hepatitis D virus infection in the global population: a systematic review and meta-analysis. Gut 68:512–521. doi:10.1136/gutjnl-2018-316601 30228220

[4] Rizzetto M , Canese MG , Aricò S , Crivelli O , Trepo C , Bonino F , Verme G . 1977. Immunofluorescence detection of new antigen-antibody system (delta/anti-delta) associated to hepatitis B virus in liver and in serum of HBsAg carriers. Gut 18:997–1003. doi:10.1136/gut.18.12.997 75123 PMC1411847

[5] Ni Y , Lempp FA , Mehrle S , Nkongolo S , Kaufman C , Fälth M , Stindt J , Königer C , Nassal M , Kubitz R , Sültmann H , Urban S . 2014. Hepatitis B and D viruses exploit sodium taurocholate co-transporting polypeptide for species-specific entry into hepatocytes. Gastroenterology 146:1070–1083. doi:10.1053/j.gastro.2013.12.024 24361467

[6] Mentha N , Clément S , Negro F , Alfaiate D . 2019. A review on hepatitis D: from virology to new therapies. J Adv Res 17:3–15. doi:10.1016/j.jare.2019.03.009 31193285 PMC6526199

[7] Perez-Vargas J , Amirache F , Boson B , Mialon C , Freitas N , Sureau C , Fusil F , Cosset F-L . 2019. Enveloped viruses distinct from HBV induce dissemination of hepatitis D virus in vivo. Nat Commun 10:2098. doi:10.1038/s41467-019-10117-z 31068585 PMC6506506

[8] Weller ML , Gardener MR , Bogus ZC , Smith MA , Astorri E , Michael DG , Michael DA , Zheng C , Burbelo PD , Lai Z , Wilson PA , Swaim W , Handelman B , Afione SA , Bombardieri M , Chiorini JA . 2016. Hepatitis delta virus detected in salivary glands of sjögren's syndrome patients and recapitulates a sjögren's syndrome-like phenotype in vivo. Pathog Immun 1:12–40. doi:10.20411/pai.v1i1.72 27294212 PMC4902173

[9] Hetzel U , Szirovicza L , Smura T , Prähauser B , Vapalahti O , Kipar A , Hepojoki J . 2019. Identification of a novel Deltavirus in boa constrictors. mBio 10:e00014-19. doi:10.1128/mBio.00014-19 30940697 PMC6445931

[10] Wille M , Netter HJ , Littlejohn M , Yuen L , Shi M , Eden J-S , Klaassen M , Holmes EC , Hurt AC . 2018. A divergent hepatitis D-like agent in birds. Viruses 10:720. doi:10.3390/v10120720 30562970 PMC6315422

[11] Maya S , Ploss A . 2020. Master of disguise: hepatitis delta virus packaging and spread facilitated by diverse viral envelope proteins. Hepatology 71:380–382. doi:10.1002/hep.30922 31465549

[12] Roggenbach I , Chi X , Lempp FA , Qu B , Walter L , Wu R , Gao X , Schnitzler P , Ding Y , Urban S , Niu J . 2021. HDV seroprevalence in HBsAg-positive patients in China occurs in hotspots and is not associated with HCV mono-infection. Viruses 13:1799. doi:10.3390/v13091799 34578380 PMC8473203

[13] Cappy P , Lucas Q , Kankarafou N , Sureau C , Laperche S . 2021. No evidence of hepatitis C virus (HCV)-assisted hepatitis D virus propagation in a large cohort of HCV-positive blood donors. J Infect Dis 223:1376–1380. doi:10.1093/infdis/jiaa517 32804999

[14] Negro F . 2014. Hepatitis D virus coinfection and superinfection. Cold Spring Harb Perspect Med 4:a021550. doi:10.1101/cshperspect.a021550 25368018 PMC4208707

[15] Fattovich G , Giustina G , Christensen E , Pantalena M , Zagni I , Realdi G , Schalm SW . 2000. Influence of hepatitis delta virus infection on morbidity and mortality in compensated cirrhosis type B. Gut 46:420–426. doi:10.1136/gut.46.3.420 10673308 PMC1727859

[16] Sagnelli C , Sagnelli E , Russo A , Pisaturo M , Occhiello L , Coppola N . 2021. HBV/HDV co-infection: epidemiological and clinical changes, recent knowledge and future challenges. Life (Basel) 11:169. doi:10.3390/life11020169 33671730 PMC7926847

[17] Yan H , Zhong G , Xu G , He W , Jing Z , Gao Z , Huang Y , Qi Y , Peng B , Wang H , Fu L , Song M , Chen P , Gao W , Ren B , Sun Y , Cai T , Feng X , Sui J , Li W . 2012. Sodium taurocholate cotransporting polypeptide is a functional receptor for human hepatitis B and D virus. Elife 1. doi:10.7554/eLife.00049 PMC3485615 23150796

[18] He W , Cao Z , Mao F , Ren B , Li Y , Li D , Li H , Peng B , Yan H , Qi Y , Sun Y , Wang F , Sui J , Li W , Ou J-HJ . 2016. Modification of three amino acids in sodium taurocholate cotransporting polypeptide renders mice susceptible to infection with hepatitis D virus in vivo . J Virol 90:8866–8874. doi:10.1128/JVI.00901-16 27466423 PMC5021397

[19] Li H , Zhuang Q , Wang Y , Zhang T , Zhao J , Zhang Y , Zhang J , Lin Y , Yuan Q , Xia N , Han J . 2014. HBV life cycle is restricted in mouse hepatocytes expressing human NTCP. Cell Mol Immunol 11:175–183. doi:10.1038/cmi.2013.66 24509445 PMC4003384

[20] Baron JL , Gardiner L , Nishimura S , Shinkai K , Locksley R , Ganem D . 2002. Activation of a nonclassical NKT cell subset in a transgenic mouse model of hepatitis B virus infection. Immunity 16:583–594. doi:10.1016/s1074-7613(02)00305-9 11970881

[21] Lempp FA , Qu B , Wang YX , Urban S . 2016. Hepatitis B virus infection of a mouse hepatic cell line reconstituted with human sodium taurocholate cotransporting polypeptide. J Virol 90:4827–4831. doi:10.1128/JVI.02832-15 26865711 PMC4836309

[22] He W , Ren B , Mao F , Jing Z , Li Y , Liu Y , Peng B , Yan H , Qi Y , Sun Y , Guo JT , Sui J , Wang F , Li W , Siddiqui A . 2015. Hepatitis D virus infection of mice expressing human sodium taurocholate co-transporting polypeptide. PLoS Pathog 11:e1004840. doi:10.1371/journal.ppat.1004840 25902143 PMC4406467

[23] Winer BY , Shirvani-Dastgerdi E , Bram Y , Sellau J , Low BE , Johnson H , Huang T , Hrebikova G , Heller B , Sharon Y , Giersch K , Gerges S , Seneca K , Pais MA , Frankel AS , Chiriboga L , Cullen J , Nahass RG , Lutgehetmann M , Toettcher JE , Wiles MV , Schwartz RE , Ploss A . 2018. Preclinical assessment of antiviral combination therapy in a genetically humanized mouse model for hepatitis delta virus infection. Sci Transl Med 10:eaap9328. doi:10.1126/scitranslmed.aap9328 29950446 PMC6337727

[24] Giersch K , Hermanussen L , Volz T , Volmari A , Allweiss L , Sureau C , Casey J , Huang J , Fischer N , Lütgehetmann M , Dandri M . 2021. Strong replication interference between hepatitis delta viruses in human liver chimeric mice. Front Microbiol 12:671466. doi:10.3389/fmicb.2021.671466 34305837 PMC8297590

[25] Ohashi K , Marion PL , Nakai H , Meuse L , Cullen JM , Bordier BB , Schwall R , Greenberg HB , Glenn JS , Kay MA . 2000. Sustained survival of human hepatocytes in mice: a model for in vivo infection with human hepatitis B and hepatitis delta viruses. Nat Med 6:327–331. doi:10.1038/73187 10700236

[26] Lütgehetmann M , Mancke LV , Volz T , Helbig M , Allweiss L , Bornscheuer T , Pollok JM , Lohse AW , Petersen J , Urban S , Dandri M . 2012. Humanized chimeric uPA mouse model for the study of hepatitis B and D virus interactions and preclinical drug evaluation. Hepatology 55:685–694. doi:10.1002/hep.24758 22031488

[27] Giersch K , Helbig M , Volz T , Allweiss L , Mancke LV , Lohse AW , Polywka S , Pollok JM , Petersen J , Taylor J , Dandri M , Lütgehetmann M . 2014. Persistent hepatitis D virus mono-infection in humanized mice is efficiently converted by hepatitis B virus to a productive co-infection. Journal of Hepatology 60:538–544. doi:10.1016/j.jhep.2013.11.010 24280293

[28] Schulze A , Schieck A , Ni Y , Mier W , Urban S . 2010. Fine mapping of pre-S sequence requirements for hepatitis B virus large envelope protein-mediated receptor interaction. J Virol 84:1989–2000. doi:10.1128/JVI.01902-09 20007265 PMC2812397

[29] Gripon P , Cannie I , Urban S . 2005. Efficient inhibition of hepatitis B virus infection by acylated peptides derived from the large viral surface protein. J Virol 79:1613–1622. doi:10.1128/JVI.79.3.1613-1622.2005 15650187 PMC544121

[30] Pearson T , Shultz LD , Miller D , King M , Laning J , Fodor W , Cuthbert A , Burzenski L , Gott B , Lyons B , Foreman O , Rossini AA , Greiner DL . 2008. Non-obese diabetic-recombination activating gene-1 (NOD-Rag1 null) interleukin (IL)-2 receptor common gamma chain (IL2r gamma null) null mice: a radioresistant model for human lymphohaematopoietic engraftment. Clin Exp Immunol 154:270–284. doi:10.1111/j.1365-2249.2008.03753.x 18785974 PMC2612717

[31] Dahari H , Feliu A , Garcia-Retortillo M , Forns X , Neumann AU . 2005. Second hepatitis C replication compartment indicated by viral dynamics during liver transplantation. J Hepatol 42:491–498. doi:10.1016/j.jhep.2004.12.017 15763335

[32] Qiao L , Luo GG . 2019. Human apolipoprotein E promotes hepatitis B virus infection and production. PLoS Pathog 15:e1007874. doi:10.1371/journal.ppat.1007874 31393946 PMC6687101

[33] Ishida Y , Chung TL , Imamura M , Hiraga N , Sen S , Yokomichi H , Tateno C , Canini L , Perelson AS , Uprichard SL , Dahari H , Chayama K . 2018. Acute hepatitis B virus infection in humanized chimeric mice has multiphasic viral kinetics. Hepatology 68:473–484. doi:10.1002/hep.29891 29572897 PMC6097938

[34] Netter HJ , Kajino K , Taylor JM . 1993. Experimental transmission of human hepatitis delta virus to the laboratory mouse. J Virol 67:3357–3362. doi:10.1128/JVI.67.6.3357-3362.1993 8497056 PMC237679

